# Molecular Diagnostics and Personalized Therapeutics in Differentiated Thyroid Carcinoma: A Clinically Oriented Review †

**DOI:** 10.3390/diagnostics15192493

**Published:** 2025-09-30

**Authors:** Andrés Coca-Pelaz, Juan Pablo Rodrigo, Mark Zafereo, Iain Nixon, Pia Pace-Asciak, Gregory W. Randolph, Carlos Suárez, Alfio Ferlito

**Affiliations:** 1 Department of Otolaryngology, Hospital Universitario Central de Asturias, University of Oviedo, ISPA, IUOPA, CIBERONC, 33011 Oviedo, Spain; jprodrigo@uniovi.es; 2 Department of Head & Neck Surgery, MD Anderson Cancer Center, Houston, TX 77030, USA; mzafereo@mdanderson.org; 3 Departments of Surgery and Otolaryngology, Head and Neck Surgery, Edinburgh University, Edinburgh EH8 9YL, UK; iain.nixon@edinburghent.com; 4 Department of Otolaryngology—Head and Neck Surgery, St. Joseph’s Health Centre, University of Toronto, Toronto, ON M5S 1A1, Canada; 5 Division of Thyroid and Parathyroid Endocrine Surgery, Department of Otolaryngology, Massachusetts Eye and Ear Infirmary, Boston, MA 02114, USA; gregory_randolph@meei.harvard.edu; 6 Instituto de Investigación Sanitaria del Principado de Asturias, IUOPA, CIBERONC, 33011 Oviedo, Spain; csuareznieto@gmail.com; 7 Coordinator of the International Head and Neck Scientific Group, 35100 Padua, Italy; profalfioferlito@gmail.com

**Keywords:** differentiated thyroid carcinoma, molecular diagnostics, precision medicine, thyroid nodule, targeted therapy, risk stratification

## Abstract

Differentiated thyroid carcinoma (DTC) is the most common endocrine malignancy and typically has a favorable prognosis. However, a subset of patients experience aggressive disease, recurrence, or treatment resistance, underscoring the need for more precise diagnostic and therapeutic strategies. Advances in molecular profiling have improved the management of thyroid cancer by enabling risk-adapted treatment and targeted interventions. This narrative review offers a clinically focused synthesis of the current role of molecular diagnostics and personalized therapeutics in DTC. We examine key genetic alterations and their diagnostic, prognostic, and therapeutic implications, and discuss how molecular markers enhance traditional risk stratification systems, informing surgical decisions, radioactive iodine (RAI) use, and surveillance. The growing role of targeted therapies, such as tyrosine kinase inhibitors and agents against specific oncogenic drivers, is reviewed, particularly for RAI-refractory DTC. We also address real-world challenges in implementing precision medicine, including access, cost, and standardization. Future directions, such as liquid biopsy, artificial intelligence, and multi-omic integration, are explored as tools to achieve fully personalized care. This review aims to bridge the gap between molecular discovery and clinical application, offering practical insights for endocrinologists, surgeons, oncologists, and multidisciplinary teams managing DTC.

## 1. Introduction

Thyroid cancer represents the most common endocrine malignancy, with the observed increase in incidence largely attributed to improved detection methods rather than a true rise in disease occurrence [1]. Differentiated thyroid cancer (DTC), which includes papillary thyroid carcinoma (PTC), follicular thyroid carcinoma (FTC), and oncocytic (Hürthle cell) thyroid carcinoma, constitutes the predominant histological category. Most DTCs are well-differentiated and generally associated with a favorable prognosis. Contemporary management has shifted toward a more individualized approach, with lobectomy being sufficient for a substantial proportion of patients, while total thyroidectomy and radioactive iodine (RAI) ablation are now reserved for selected cases, and thyroid-stimulating hormone (TSH) suppression is applied when clinically indicated [2].

Despite the generally favorable prognosis of DTC, challenges exist with traditional diagnostic and therapeutic approaches. Thyroid nodules are very common, but only 5–15% are malignant [3]. Fine needle aspiration (FNA) cytology is a primary diagnostic tool, but approximately 20–25% of aspirates result in indeterminate cytology, with a malignancy risk ranging from 6% to 40% [4,5]. Historically, surgery was recommended for definitive diagnosis of indeterminate thyroid nodules (ITNs). This was due to difficulty in differentiating benign from malignant nodules using radiology and cytology alone. This led to unnecessary invasive procedures in up to 75% of such cases, which were benign on final histopathology [6]. Furthermore, while overall mortality in DTC is low, about 15% of cases are locally invasive, and those with distant metastases refractory to RAI treatment have a particularly poor prognosis. For more aggressive subtypes such as poorly differentiated and anaplastic thyroid cancer, disease-specific mortality is high, highlighting the need for more effective treatments beyond traditional therapies [6,7].

The rise of precision medicine is transforming the management of thyroid cancer. Personalized medicine aims to define patients based on their unique genetic information to select treatments with optimal efficacy and predict disease behavior or risk. Advances in sequencing and integrated genomics have provided new insights into the genetic lesions and modifications responsible for thyroid cancer onset, progression, dedifferentiation, and metastasis [8]. Molecular analysis, including the study of genetic mutations, non-coding RNAs, and chromatin remodeling, is changing the landscape of thyroid cancer research and care. Molecular diagnostics can improve diagnostic accuracy for ITNs and provide actionable information regarding tumor prognosis. Identification of targetable alterations, such as mutations in *BRAF*, *RAS*, *RET*, and gene fusions, is crucial for targeted systemic therapies, especially in advanced, aggressive, or RAI-refractory (RR) disease [8,9].

This narrative review aims to summarize current advances in the genomic and epigenomic profile of thyroid cancer and molecular diagnostics, highlighting their impact on personalized therapies and presenting these developments within a clinically applicable framework.

## 2. Molecular Diagnostics in DTC

The diagnosis and management of thyroid cancer, particularly DTC, have been significantly influenced by advancements in understanding its molecular pathogenesis. Genetic mutations and epigenetic alterations drive thyroid tumorigenesis and progression [7]. Molecular diagnostic testing, often performed on FNA samples, has emerged as a crucial tool to improve diagnostic accuracy, particularly for cases with indeterminate cytology, and to provide prognostic information [10].

### 2.1. Overview of Key Genetic Alterations

Several key genetic alterations are frequently observed in thyroid cancer, primarily affecting signaling pathways such as the MAPK and PI3K pathways [11]. Detection of these alterations is used in molecular diagnostic panels [12]. Some of the most significant include the following:

- ***BRAF* Mutations**: Mutations in the *BRAF* gene are common in PTC [13]. The BRAF V600E mutation is the most extensively studied variant. It is highly specific for malignancy, particularly PTC, and is rare in FTC or benign nodules. When detected on FNA, even in indeterminate or non-diagnostic samples, a diagnosis of thyroid cancer is strongly suspected [3,14]. While BRAF V600E is the most frequent, other *BRAF* variants and translocations also occur, especially in follicular-patterned tumors.
- ***RAS* Mutations**: Mutations in the RAS gene family, including HRAS, KRAS, and NRAS, are found in both benign and malignant thyroid lesions [11]. In thyroid cancer, RAS mutations are more commonly associated with FTC and can also be detected, albeit at lower frequencies (approximately 10% or less), in the follicular variant of PTC [15]. These genes encode proto-oncogenes involved in key signaling pathways regulating cell proliferation and differentiation.
- ***RET* Fusions (RET/PTC)**: RET gene alterations, most commonly referred to as RET fusions, involve the fusion of the RET proto-oncogene—encoding a receptor tyrosine kinase—with various partner genes [16]. These fusions are characteristic genetic events in PTC. Inherited RET point mutations, in contrast, are associated with medullary thyroid carcinoma (MTC) [17].
- ***TERT* Promoter Mutations**: Mutations in the promoter region of the *TERT* gene (telomerase reverse transcriptase) are significant alterations. *TERT* promoter mutations are reported in DTC and are frequently found coexisting with *BRAF* or *RAS* mutations. These mutations are often associated with more aggressive disease across thyroid carcinoma subtypes and may serve as a marker of poor prognosis [18].

Other significant genetic alterations include rearrangements like PAX8/PPAR gamma fusion (*PAX8*, *PPARG*), particularly frequent in FTC and FVPTC [19], and mutations in genes involved in the PI3K pathway, such as PIK3CA, PTEN, and AKT1 [20]. Mutations in tumor suppressor genes like *TP53* are also important, especially in poorly differentiated and anaplastic thyroid carcinomas. Gene fusions involving NTRK and ALK are also recognized [21].

### 2.2. Role of Molecular Testing in Cytologically ITNs

FNA cytology is the gold standard for assessing thyroid nodules [22]. However, a significant proportion of samples (about 15–30%) yield indeterminate results, falling into Bethesda categories III (Atypia of Unknown Significance/Follicular Lesion of Unknown Significance) and IV (Follicular Neoplasm) [23]. Although the Bethesda System for Reporting Thyroid Cytopathology (TBSRTC) also classifies Bethesda V (Suspicious for malignancy) as indeterminate, this category carries a substantially higher risk of malignancy and is therefore usually managed surgically. For this reason, Bethesda V is typically omitted when discussing molecular testing in ITNs. The Risk of Malignancy (ROM) associated with these cytological categories exceeded thresholds considered acceptable for conservative management, thereby warranting surgical intervention. Molecular testing has become a commonly utilized tool to provide additional risk information for these ITNs. The goal is to shift the ROM for ITNs from around 25% to levels of risk that help determine whether conservative surveillance or surgical intervention is more appropriate [24,25]. By identifying specific genetic alterations, molecular testing can significantly improve the diagnostic accuracy beyond cytology alone.

### 2.3. Commercially Available Tests: Performance, Strengths, and Limitations

Several commercially available molecular tests are routinely used to evaluate FNA samples, particularly from ITNs. These platforms employ different molecular methodologies but share the common goal of refining the ROM and aiding in clinical decision-making [6]. These assays use various genomic technologies to assess the risk of malignancy and inform clinical decision-making. Among the most widely used in the United States are Afirma GSC, ThyroSeq v3, the combined ThyGeNEXT/ThyraMIR (MPTX) platform, ThyroidPrint, and mir-THYpe. While each test differs in methodology, the common goal is to provide clinicians with actionable information that goes beyond cytology alone. An overview of their key features, technologies, and clinical applications is summarized in Table 1. These tests have shown promise in improving diagnostic accuracy. For instance, a molecular classification system based on three specific CpG markers (*MBNL2*, *NPC2*, *GPCPD1*) showed significant discrimination between benign and malignant samples in follicular-patterned thyroid samples, with high sensitivity (97%) and specificity (88%) for malignancy detection in a validation cohort [26]. Molecular panels testing for mutations like *TERT*, *BRAF*, *PAX8/PPARγ*, *RAS*, and *RET/PTC* in indeterminate FNA samples can provide a strong indication for malignancy [12]. Combining molecular marker expression analysis with mutation evaluation may significantly improve diagnostic accuracy in indeterminate cases. Molecular diagnostic assays are often classified according to their clinical utility as either “rule-in” tests, which support the confirmation of malignancy, or “rule-out” tests, which aim to reliably exclude it [27]. Tests like Afirma GSC, Thyroseq v3, and mir-THYpe have been noted to perform well as “rule out” tests for ITNs. In addition, ThyroidPrint, a 10-gene expression classifier validated in prospective multicenter studies in Latin America, has emerged as a cost-effective “rule-out” tool for Bethesda III/IV nodules, showing a high negative predictive value (~95%) and reducing unnecessary surgeries [28,29].

A key strength of molecular testing is its ability to improve the pre-operative diagnosis of ITNs, potentially reducing unnecessary surgeries. The detection of specific mutations is highly predictive of malignancy. Testing for a panel of mutations in ITNs can provide a strong indication for malignancy, favoring the choice of surgery [10,30].

However, the utility of some markers, like BRAF V600E alone as a diagnostic marker for ITNs specifically, is limited by its low sensitivity, as it is infrequent in ITNs (<10%) [31]. The widespread adoption of these molecular markers is not universal outside of the United States. Implementation can be limited in some regions of Canada, South America, and Europe, often lacking national healthcare or insurance support [24,25]. In some areas, molecular diagnostics for sporadic thyroid cancer are generally not supported by regional health systems. Furthermore, there are currently no established guidelines for management decisions based on most genetic alterations detected in thyroid nodules, regardless of cytology category [6]. Health economic analysis of these tests varies in outcome, in part at least due to the relative costs of healthcare in different countries. Although molecular testing is expensive, its cost can be offset by avoiding diagnostic lobectomies. As the price of molecular platforms continues to decrease, their availability and use are expected to expand to a broader patient population.

**Table 1:** Comparison of commonly used molecular tests for ITNs.

Test	Platform/Technology	Analytes	Output	Key Features	Validation Type [Ref]
Afirma GSC	Whole-transcriptome RNA sequencing + Machine Learning	mRNA expression	Benign/Suspicious	High NPV; reclassifies Bethesda III/IV nodules	Prospective, blinded, multicenter study [32]
ThyroSeq v3	Targeted next-generation DNA/RNA sequencing	Point mutations, gene fusions, CNVs, gene expression (112 genes)	Detailed genomic profile	Diagnostic, prognostic, and therapeutic information	Prospective, blinded, multicenter validation study [33]
MPTX (ThyGeNEXT/ThyraMIR)	Targeted DNA sequencing + microRNA risk classifier	Gene mutations/rearrangements (DNA) + miRNA expression (RNA)	Benign/Moderate/High Risk score	Multiplatform approach combining DNA and microRNA analysis	Multicenter, blinded validation + real-world studies [34]
ThyroidPrint	q-PCR-based ten-gene classifier	Gene expression (RNA)	Benign/Suspicious	High NPV; reclassifies Bethesda III/IV nodules	Multicenter validation [29]
mir-THYpe	microRNA qPCR profiling on FNA cytology slides (no repeat aspiration required)	Panel of 11 microRNAs	Benign vs. malignant	High sensitivity (~95%) and specificity (~81%); high NPV (~96%); avoids unnecessary surgeries; uses existing cytology slides	Initial development and FNA-smear validation and a large real-world prospective multicenter validation [35,36]

CNVs: Copy Number Variations, NPV: Negative Predictive Value.

### 2.4. Diagnostic Implications and Clinical Decision-Making

The identification of specific genetic and epigenetic alterations has profound diagnostic and clinical implications. Beyond initial diagnosis, molecular testing is increasingly valuable for predicting prognosis and identifying potential targets for personalized therapy [11,37]. Specific mutations and fusions are associated with increased tumor aggressiveness, metastatic spread, and reduced response to standard treatments like RAI [8]. For example, *TERT* promoter mutations are considered strong markers of poor prognosis [7].

Furthermore, identifying specific genetic alterations in advanced or aggressive thyroid cancers can inform eligibility for targeted systemic therapies [38]. This is particularly important for the subset of patients with metastatic, progressive, or RR-DTC, where standard treatments are not effective. Understanding the molecular landscape allows clinicians to choose treatments targeting specific aberrant signaling pathways. The identification of targetable alterations has potential as a promising application of this technology in aggressive disease settings.

Molecular testing is transforming patient care by moving towards a personalized and precision medicine approach based on genetic information. It is advised that molecular testing be performed only if it has implications for treatment. Future research is needed to better define the clinical utility of molecular information in guiding management decisions, including the extent of surgery and prediction of tumor recurrence [39].

## 3. Integration of Molecular Data into Risk Stratification

Risk stratification plays a central role in the management of DTC, guiding prognosis and therapeutic decisions. Traditional systems like the AJCC/TNM classification (used to predict mortality) and the ATA recurrence risk model (for predicting the likelihood of recurrence) rely on clinicopathological factors such as age, tumor size, extrathyroidal extension, lymph node involvement, and distant metastases [40,41]. Recent ATA guidelines have begun incorporating molecular profiling, particularly in low-risk patients. Additionally, dynamic risk stratification (DRS) refines prognosis over time by incorporating response to initial therapy [42].

Although current guidelines do not generally recommend molecular testing for initial risk stratification, BRAF and TERT promoter mutations are included in recurrence risk stratification. The feasibility of analyzing mutations in FNA samples has been demonstrated [43].

Molecular data can significantly enhance prognostic accuracy. More comprehensive genetic analysis can lead to increasingly precise predictions of patient outcome. TERT promoter mutations are strong independent prognostic markers in thyroid cancer, and their coexistence with BRAF or RAS mutations enhances risk prediction beyond current ATA or TNM systems. Preoperative detection of TERT alterations helps identify more aggressive DTC and improves risk stratification [44,45,46]. Other studies suggest that tumors with mutation combinations such as TERTp and BRAF V600E or TERTp and RAS tend to follow a more aggressive clinical course [7,9,47,48]. Mutations in genes such as TP53, PIK3CA, AKT1, or the TERT promoter are relatively common in advanced tumors [7,49]. Specifically, TP53 (25–30%), PIK3CA (10–20%), CTNNB1 (10–20%), and AKT1 (5–10%) have been identified as markers of aggressive behavior and tumor dedifferentiation [17,50,51,52,53,54,55,56,57,58].

Classification of molecular variants and fusions into categories such as BRAF-like, RAS-like, and non-BRAF non-RAS-like has been helpful in grouping molecular alterations that share similar risks for events such as extrathyroidal extension and lymph node metastasis [59,60]. Molecular profiling has already shaped the 5th edition (2022) of the WHO Classification of Endocrine and Neuroendocrine Tumors [61], refining the categorization of thyroid neoplasms and supporting more precise risk stratification and therapeutic decisions. For instance, BRAF-like mutated tumors have shown significantly higher rates of T4 tumor size and N1b nodal metastasis (22%) compared to other classes (≤6%), among other more aggressive findings [62]. Molecular tests such as Afirma GSC, MPTX, and TSv3 have shown potential to predict disease recurrence in thyroid cancers and nodules classified as Bethesda V/VI, based on detecting high- or low-risk genetic mutations [63,64].

Beyond genomic alterations, the analysis of epigenetic modifications, such as DNA methylation, histone acetylation, and non-coding RNAs, has contributed to discovering new regulatory mechanisms of cell malignancy, particularly in aggressive forms of thyroid cancer [9].

The use of molecular findings allows tailoring both surveillance and treatment intensity [22]. TERT promoter mutation may be useful in the future to individualize treatments, such as the type of surgery and RAI therapy, as well as the intensity of patient follow-up [65].

## 4. Personalized Therapeutic Approaches

The concept of “personalized medicine” involves stratifying individuals based on their genetic information to assign different treatment approaches and predict treatment response efficacy, disease behavior, or disease risk [8,66]. In contrast to standard chemotherapy, targeted therapies aim at one or more defined molecular pathways in cancer cells, so their selection is based on the patient’s genetic information [67]. Understanding the molecular landscape of thyroid cancer is therefore crucial to providing individualized targeted therapies.

Personalized therapeutic approaches in DTC are increasingly guided by molecular findings, which influence surgical decision-making (e.g., extent of thyroidectomy), RAI use, and eligibility for targeted therapies such as TKIs and selective inhibitors [51,68]. Dabrafenib, a selective inhibitor of mutated BRAF forms, can improve RAI uptake in patients with RR metastatic PTC carrying the BRAFV600E mutation [8]. Increased efficacy of larotrectinib (LOXO-101), a selective tropomyosin receptor kinase (TRK) inhibitor, is suggested in patients with NTRK fusions [69]. Amplification of CD274, PDCD1LG2, JAK2, and DNA mismatch repair (MMR) deficiencies has been associated with a positive response to immune checkpoint inhibitors like pembrolizumab and nivolumab [70]. Molecular testing thus provides diagnostic, prognostic, and therapeutic value, making thyroid cancer a model for precision oncology.

### 4.1. Surgical Decision-Making Guided by Molecular Profile

The incorporation of molecular markers into risk stratification systems available preoperatively seems conceptually attractive to tailor patient management in terms of the initial extent of surgery, adjuvant therapy, and postoperative medical management. Molecular profiling of FNA samples can improve diagnostic accuracy in the indeterminate setting (indeterminate cytology) and support a more personalized and effective management of thyroid cancer [71]. While the extent of thyroidectomy has traditionally been guided by tumor size, location, and risk of recurrence, molecular profiling is emerging as an additional tool in preoperative risk stratification. Mutations such as BRAF V600E and TERT promoter have been associated with more aggressive disease features [72], influencing the decision to opt for total thyroidectomy over lobectomy in select cases. Conversely, the absence of high-risk mutations in ITNs may support a more conservative surgical approach. Vignali et al. [73] conducted a retrospective study analyzing ITNs with negative molecular testing for mutations such as BRAF and RAS. The findings indicated a low risk of malignancy in these cases, suggesting that limited surgery, -or even active surveillance, may be appropriate alternatives to total thyroidectomy. Furthermore, as part of the treatment algorithm for low-risk thyroid disease, such as indeterminate cytology in the absence of concerning molecular, clinical, or sonographic features, Issa et al. demonstrated that minimally invasive procedures such as radiofrequency ablation are a safe alternative option to surgery and active surveillance [74].

### 4.2. Use of RAI: Indications, Refractoriness, and Molecular Predictors of Response

DTC cells express sodium/iodide symporter (NIS) after TSH stimulation, allowing RAI to be taken up by follicular-derived thyroid cells. RAI is therefore taken up by these cells and induces cell death by low-dose irradiation [43]. RAI is considered one of the first examples of theranostics in oncology and a distinctive example of personalized medicine widely used for DTC management [75]. However, approximately 65% of patients with distant metastases ultimately develop RR disease [76,77]. RAI-refractory DTC (RR-DTC) is defined, according to the 2025 ATA guidelines [22], as disease that either (i) does not concentrate RAI on initial therapy, (ii) loses the ability to take up RAI after previous evidence of avidity, (iii) shows heterogeneity with some lesions no longer concentrating RAI, or (iv) progresses despite significant RAI uptake. This subset of patients represents the primary indication for systemic targeted therapies. This RR state is related to the NIS, also known as SLC5A5 [9]. Loss of iodine avidity in DTC can be related to genetic and epigenetic alterations and the MAPK and PI3K-AKT signaling pathways [78,79]. Dedifferentiation is related to a decrease or loss of NIS expression and/or its targeting to the plasma membrane, resulting in loss of iodine uptake in thyroid cells. The concept of a redifferentiation strategy has emerged with the purpose of finding drugs capable of restoring RAI sensitivity in refractory thyroid cancers [80]. Dabrafenib, a selective inhibitor of mutated BRAF forms, has been shown to promote RAI uptake in patients with metastatic BRAFV600E-mutated and RR PTC [81]. Other compounds, such as PI3K/AKT and MEK/ERK inhibitors, have been recommended for NIS overexpression and have been associated with improvement in RAI uptake in vitro and in vivo studies of thyroid cancers [11,82].

Certain genetic mutations may be predictors of refractoriness. For example, the TERT promoter mutation can predict RAI refractoriness in distantly metastatic DTC [49,83,84].

### 4.3. Targeted Therapies

New medications are in development that specifically target critical molecules responsible for tumor formation, known as “targeted therapy” [8]. Unlike general chemotherapy drugs, targeted therapy drugs can aim at one or more specific molecular pathways in cancer cells. In advanced and aggressive DTC, where identifying targetable mutations can have a significant clinical impact, there has been a substantial expansion of the therapeutic arsenal with genome-targeted therapies over the last decade [85,86,87,88].

Innovative approaches based on personalized medicine for thyroid cancer treatment include tyrosine kinase inhibitors (TKIs) and small molecules targeting iodine reuptake pathways.

#### 4.3.1. TKIs

Molecular alterations provide the rationale for the use of multi-target TKIs and selective inhibitors in advanced DTC [6]. These alterations have paved the way for the development of TKIs, which act on specific oncogenic pathways, signaling kinases, and angiogenic mechanisms [8]. Generally, TKIs work by blocking the action of tyrosine kinase enzymes, which play a critical role in cell signaling, growth, and division. Non-specific TKIs such as Motesanib, Sunitinib, Sorafenib, and Lenvatinib, along with VEGF receptor-targeting agents like Axitinib, have demonstrated efficacy in advanced disease [85,86,89,90,91]. Cabozantinib is another multi-target TKI used in this context [92]. More recently, selective RET inhibitors such as BLU-667 (Pralsetinib) and Selpercatinib (LOXO-292) have demonstrated promising activity in RET-altered thyroid cancers [93,94,95,96,97]. BLU-667 inhibits the protein product of RETM918T, as well as RETV804L/M gatekeeper mutations conferring resistance to TKIs, while LOXO-292 is a highly selective RET kinase inhibitor with nanomolar potency against the canonical RET MTC drivers, RET gatekeeper mutations, and RET fusions [98]. These targeted therapies specifically inhibit RET alterations found in tumors like MTC and PTC. Clinical trials, such as the ARROW trial for Pralsetinib [96] and LIBRETTO-001 for Selpercatinib [93], have shown significant objective response rates in patients with RET-mutant tumors. This promising efficacy has led to the FDA approval of both Pralsetinib and Selpercatinib for advanced or metastatic RET-altered cancers, including MTC [97].

#### 4.3.2. Novel Agents Targeting MEK, NTRK, BRAF

More specific agents have also been developed; Vemurafenib is an inhibitor of the BRAF V600E mutation, which disrupts the MAPK signaling pathway involved in the uncontrolled growth of cancer cells [99]. Selumetinib is a selective MEK inhibitor, which inhibits the MEK enzyme that is part of the RAS signaling pathway and an inducer of iodine reuptake [100]. Detailed knowledge of mutations, fusions, and gene expression profiles will drive the discovery and development of new drugs. Increased efficacy of Larotrectinib (LOXO-101) as a selective tropomyosin receptor kinase (TRK) inhibitor has been suggested in patients with NTRK fusions [69,101].

### 4.4. Criteria for Initiating Targeted Therapy and Monitoring Response

Systemic targeted therapy with TKIs is generally indicated for patients with RR DTC exhibiting structural disease progression that poses clinical risk. International guidelines and clinical trials recommend TKI initiation when metastases are measurable and demonstrate significant growth over time, typically defined as a reliable increase in lesion size (e.g., doubling time or RECIST-defined progression) or rapid structural advancement, often referred to as the “inflection point” for intervention [102,103,104].

Additional criteria include the following:

- Confirmed RR, usually established by cumulative RAI activity exceeding ~600 mCi or insufficient RAI uptake despite prior therapy [105].
- Measurable tumor burden with imaging evidence of progression, symptomatic disease, or threat to vital structures [102,104].
- Overall patient performance status (e.g., ECOG 0–2), adequate organ function, and comorbidity assessment to ensure tolerability [102,106].

Once therapy is initiated, response monitoring should integrate multiple modalities, as follows:

1. Radiologic assessment using standardized criteria such as RECIST 1.1, typically via CT or MRI every 8–12 weeks to quantify changes in target lesion dimensions [103,107].
2. Serum thyroglobulin (Tg) levels serve as a biochemical marker of treatment efficacy when anti-Tg antibodies are absent [103].
3. Clinical evaluation of symptoms, performance status, and treatment-related toxicities (e.g., hypertension, hand–foot syndrome, diarrhea) to guide dose modification [105,108].
4. In selected cases, especially where cross-sectional imaging is ambiguous, functional imaging (e.g., FDG-PET or RAI scans) or emerging modalities (liquid biopsy, tumor mutation burden) can provide additional insight [102,103].

Clear early identification of disease progression, coupled with vigilant biochemical and clinical monitoring, allows prompt adaptation of therapy (including dose adjustments, treatment breaks, or transition to next-line agents), thus optimizing outcomes while balancing toxicity.

### 4.5. Toxicity Management and Patient Selection

Systemic therapy with TKIs such as Sorafenib, Lenvatinib, and Cabozantinib is integral to treating progressive RR DTC, but these agents are frequently associated with adverse events that require careful management to maintain treatment benefit and patient quality of life.

#### 4.5.1. Adverse Event Profile and Monitoring

In the DECISION trial [86], Sorafenib was associated with a high incidence of adverse events (AEs), with nearly all patients (98.6%) experiencing at least one AE. The most frequent events included hand-foot skin reaction (HFSR) in 76% of patients, diarrhea in 69%, alopecia in 67%, and rash in 50% of cases. Similarly, in the SELECT trial [109], Lenvatinib demonstrated a distinct AE profile, with diarrhea and fatigue both occurring in 67% of patients, followed by proteinuria (32%), rash (23%), and palmar–plantar erythrodysesthesia syndrome (PPES) in 33% of cases. Grade 3 AEs were primarily proteinuria (10%) and diarrhea (9%), while treatment discontinuations due to toxicity were relatively uncommon (<3%). General management of TKI-related AEs (Table 2) depends on their severity and impact on patient quality of life. Grade 1–2 AEs are typically managed with supportive measures, allowing continuation of therapy without dose modifications. In contrast, grade 3–4 AEs often necessitate temporary treatment interruption, dose reduction, or even permanent discontinuation if toxicity persists or poses a significant risk [85,86,109,110].

A multidisciplinary approach involving dermatology, cardiology, nephrology, and nutritional support is recommended to optimize management of toxicities, minimize treatment interruptions, and maintain patient quality of life.

#### 4.5.2. Management Strategies

Management of TKI-related AEs requires proactive strategies to minimize toxicity and maintain therapeutic efficacy. Early detection is essential, with weekly blood pressure measurements during the first 6–8 weeks of treatment and urinalysis every 4–6 weeks to detect proteinuria, particularly in patients receiving Lenvatinib [85,109]. Hypertension is typically managed with first-line antihypertensives such as Angiotensin-converting enzyme inhibitors or calcium-channel blockers, while persistent grade ≥ 3 hypertension often necessitates dose reduction. Dermatologic adverse events, including HFSR, are addressed through preventive skin care with emollients and, in severe cases, temporary treatment interruption following the DECISION trial guidelines [86]. Diarrhea is managed with dietary adjustments and loperamide, with treatment interruption and dose reduction required for grade ≥ 3 events. Proteinuria is monitored through serial urinalysis, and therapy should be interrupted or the dose reduced if urinary protein levels exceed 2 g/24 h or if signs of nephrotic syndrome appear [85,109].

#### 4.5.3. Patient Selection and Dose Optimization

Proper initiation of TKI therapy necessitates a thorough baseline assessment, including evaluation of functional status (ECOG 0–2), cardiovascular health, and renal and hepatic function before treatment initiation. Dose titration strategies, supported by real-world evidence and clinical trial data, often involve reducing the starting dose of Lenvatinib from 24 mg to 14–18 mg in vulnerable patient populations to maintain efficacy while improving tolerability [85,109].

This concentrated approach to toxicity management aims to prolong treatment continuity, optimize clinical outcomes, and preserve quality of life in patients receiving TKIs for RR DTC.

## 5. Clinical Implementation and Real-World Considerations

### 5.1. Challenges in Adopting Molecular Testing: Cost, Accessibility, Standardization

Despite its potential to transform the management of DTC, molecular testing faces several formidable barriers to clinical implementation, as follows:

- High cost and uncertain reimbursement: Molecular assays, particularly next-generation sequencing (NGS) panels, can cost between USD 1700 and 3500 per test [25,111,112]. While the upfront cost may be offset in some healthcare settings by reducing diagnostic surgeries (e.g., diagnostic lobectomies), cost-effectiveness varies widely across regions and reimbursement systems [113]. In many middle- and low-income countries, these tests are either not covered by public funding or require substantial out-of-pocket payments, severely limiting patient access [112].
- Limitations in laboratory infrastructure and workflow: Many institutions lack the infrastructure and technical expertise necessary for high-quality NGS diagnostics. In Canada and parts of Europe, molecular testing remains centralized in academic centers, and community hospitals rarely have access to validated testing platforms or well-trained molecular pathologists [114,115]. Furthermore, inconsistency in sample processing, analytical validation, and reporting among different laboratories leads to variable results and undermines clinical confidence [116].
- Lack of standardization and harmonized guidelines: Although professional societies, such as the European Thyroid Association, recommend molecular testing in the evaluation of ITNs, they also underscore the need for standardized protocols regarding assay choice, mutation panels, and analytic thresholds [25,115,117]. Currently, wide variability exists in testing strategies (e.g., sequential single-gene assays versus broad-panel NGS) and interpretation frameworks, making it difficult to compare outcomes or implement uniform clinical pathways [114].

In summary, the effective deployment of molecular diagnostics in thyroid cancer is impeded by high costs, limited accessibility, inconsistent laboratory workflows, and the absence of harmonized implementation standards. Addressing these challenges is critical to ensuring equitable and evidence-based use of molecular testing in everyday clinical practice.

### 5.2. Multidisciplinary Team Approach to Interpretation and Application of Results

Effective implementation of molecular testing in DTC management depends heavily on a coordinated, multidisciplinary team (MDT) approach. Consensus guidelines from the American Head and Neck Society (AHNS) and International Thyroid Oncology Group (ITOG) emphasize that optimal patient care involves endocrinologists, medical oncologists, surgeons, pathologists, radiologists, and radiation oncologists collaborating closely to interpret molecular findings in a clinical context. This multidisciplinary framework supports personalized treatment decisions and improves integration of emerging diagnostic biomarkers into practice [6,118,119,120].

Real-world data corroborate the added value of MDTs: an institutional study found that 15% of thyroid cancer cases reviewed in endocrine tumor boards resulted in management changes, especially in recurrent disease, demonstrating increased alignment with evidence-based guidelines [121]. Survey-based research further indicates that over two-thirds of specialists perceive MDTs as enhancing treatment selection, reducing variability, promoting guideline adherence, and improving both continuing education and patient satisfaction [122].

Moreover, MDTs enable rigorous evaluation of molecular data from validated laboratories (e.g., those with CLIA accreditation or equivalent), including specimen adequacy and analytical technique selection (e.g., targeted NGS panels versus single-gene tests) to ensure reliable, actionable results. This is particularly important in cases where molecular platforms differ in sensitivity, specificity, and scope of mutations or fusions tested [6,120].

Therefore, clinical interpretation and application of molecular test results in DTC are most effective when facilitated through a multidisciplinary tumor board structure, ensuring consistency in patient selection, result validity, and alignment with evolving targeted treatment algorithms.

### 5.3. Disparities in Global Availability and Use

The implementation of molecular testing in DTC remains heterogeneous worldwide, with marked disparities in access and clinical integration.

- Regional inequities: North America dominates the molecular diagnostics market, with widespread adoption in academic centers and reimbursement in many settings, although rural regions face reduced uptake due to workforce and funding limitations) [112,114,119]. In contrast, access in low- and middle-income countries is minimal, restricted by out-of-pocket costs and lack of public coverage [123].
- Uneven adoption within regions: In Europe, availability varies considerably. A multinational survey reported that while most clinicians prescribe molecular testing for aggressive thyroid cancers, barriers such as limited reimbursement, absence of standardized workflows, and restricted access to targeted therapies remain. Notably, only two-thirds of centers reported functioning molecular tumor boards [114].
- Infrastructure and economic constraints: Outside specialized institutions, inadequate laboratory capacity, personnel shortages, and quality-control gaps compromise testing reliability. Although costs are declining, cost-effectiveness remains highly context-dependent [112,114,116].

Overall, disparities are shaped by economic, infrastructural, and policy factors, underscoring the need for harmonized guidelines, resource investment, and equitable reimbursement models.

### 5.4. Insights from Real-World Data and Practice Guidelines

Real-world and health technology assessment studies consistently underscore the diagnostic and economic value of molecular testing in the management of ITNs (Bethesda III/IV).

- A decision-analytic cost-effectiveness model demonstrated that ThyroSeq v3 (TSv3) and Afirma Genomic Sequencing Classifier (GSC) are substantially more cost-efficient than upfront diagnostic lobectomies in the US healthcare setting. The cost per correct diagnosis was estimated at approximately USD 14,277 for TSv3, USD 17,873 for GSC, and USD 38,408 for lobectomy, making TSv3 the preferred strategy in robust sensitivity analyses [105,124,125].
- Another Markov model comparing reflexive versus selective molecular testing revealed an average cost of USD 8045 per patient in the reflexive strategy versus USD 6090, but resulted in fewer unnecessary lobectomies and a cost per surgery avoided of approximately USD 20,600 [126].
- A comprehensive health technology assessment found that molecular testing improved diagnostic accuracy (sensitivity), significantly reduced unnecessary surgeries (from ~75% to ~21%), and slightly increased quality-adjusted life years (QALYs), although the incremental cost-effectiveness ratio (ICER) was high (USD 220,572–298,653 per QALY), rendering it not cost-effective at traditional willingness-to-pay thresholds [124].

Regarding impact on clinical practice, a recent systematic review and meta-analysis encompassing 31 studies and 4,464 ITNs reported surgical avoidance rates from 50.3% to 68.6%, with ThyGenX/ThyraMIR achieving the highest rate at ~68.6% (95% CI 63.1–73.9%) [127]. This confirms that the use of molecular testing frequently results in decreased surgical interventions in real-world settings.

Finally, clinical practice guidelines from leading organizations (e.g., ATA, ESMO) now recommend integrating molecular profiling -particularly when actionable alterations such as BRAF V600E, RET, or NTRK fusions are detectable- into the planning for systemic therapy in patients with RR or metastatic thyroid carcinoma. These guidelines also stress the importance of interpreting results in the context of clinical, cytological, and radiological findings [119].

## 6. Future Perspectives in Precision Management

The future of precision management in DTC lies in leveraging cutting-edge technologies and multi-layered molecular data to achieve fully personalized care. Emerging tools such as liquid biopsy, AI-assisted ultrasound and cytology, and radiogenomics are poised to enhance non-invasive diagnostics and improve risk stratification. The integration of multi-omics approaches—encompassing genomics, proteomics, and transcriptomics-promises a deeper understanding of tumor heterogeneity and therapeutic vulnerabilities. Ongoing and future clinical trials are exploring how precision-guided therapies can be optimized in DTC, setting the stage for a holistic, individualized care pathway that aligns molecular profiles with tailored interventions.

### 6.1. Emerging Technologies: Liquid Biopsy, AI in Ultrasound and Cytology, Radiogenomics

Recent innovations are poised to further refine personalized management of DTC by integrating non-invasive biomarkers, artificial intelligence (AI), and imaging-genomic correlations.

- Liquid biopsy represents a promising non-invasive approach to detect circulating tumor DNA (ctDNA), circulating tumor cells (CTCs), and exosomal microRNAs in blood samples, offering real-time insights into tumor genetics, minimal residual disease, and treatment response [128,129].
- AI applied to ultrasound and cytology is revolutionizing thyroid nodule assessment. Deep learning algorithms have demonstrated diagnostic performance comparable to or superior to that of expert radiologists in stratifying nodules and reducing unnecessary biopsies, in the context of thyroid nodule risk stratification. For example, the S-Detect AI system achieved sensitivity and accuracy similar to experienced radiologists, and significantly improved diagnostic performance among trainees while lowering biopsy rates by up to 27% in a real-world prospective study [130,131].
- Radiogenomics, which integrates quantitative imaging features (radiomics) with tumor genomic signatures, has shown early potential in thyroid cancer. A recent systematic review demonstrated correlations between ultrasound CT/MRI-derived radiomic features and genomic alterations such as BRAF V600E and RET/PTC fusions, as well as prediction of nodal metastases [132].

### 6.2. Integration of Multi-Omics Approaches (Genomics, Proteomics, Transcriptomics)

The integration of multi-omics technologies, including genomics, transcriptomics, proteomics, and metabolomics, has ushered in a comprehensive understanding of thyroid cancer biology, enabling the discovery of actionable biomarkers and molecular subtypes with distinct prognostic and therapeutic implications. A recent review highlights that high-throughput omics approaches reveal the molecular complexity of thyroid tumors and offer substantial translational potential for personalized patient management [133,134,135].

Specifically, integrative analyses have delineated metabolic and molecular phenotypes linked to oncogenic mutations. One study demonstrated that BRAF-like and RAS-like DTCs exhibit unique metabolic signatures, such as enrichment of tricarboxylic acid cycle intermediates and branched-chain amino acid pathways, and identified protein markers like SLC7A5 and SHMT2 whose knockdown impaired tumor cell proliferation [135].

Broader clustering of multi-omics data (including DNA methylation, gene expression, non-coding RNA profiling, and mutation profiles) in a cohort of 539 patients resulted in the identification of two distinct molecular subtypes (CS1, CS2) with differential prognosis, immune infiltration, and predicted drug sensitivities. These findings underscore the utility of multi-omics in defining biologically relevant thyroid cancer subgroups that could inform therapy selection [136].

Furthermore, consensus machine-learning frameworks trained on multi-omics data from TCGA-THCA have yielded prognostic models capable of stratifying thyroid carcinoma patients by survival outcomes and immune status. These models have identified hub genes such as SNAI1, validated in vitro, thus linking integrated multi-omics clustering with functional tumor biology and potential therapeutic targets [133,134,137].

Multi-omics integration provides a powerful platform for capturing tumor heterogeneity, identifying subtype-specific vulnerabilities, and enabling predictive stratification. Its implementation holds promise for precision risk assessment and targeted therapy planning in DTC.

### 6.3. Current and Future Clinical Trials Exploring Precision Therapy in DTC

Several ongoing and recently completed trials are evaluating precision-guided treatments in RR-DTC, focusing on targeted therapy alone and in combination with immunotherapy or novel neoadjuvant strategies.

- A Phase II study by the International Thyroid Oncology Group (ITOG) investigated pembrolizumab combined with Lenvatinib in progressive RR DTC (NCT02973997). Accrual has concluded, with results anticipated by late 2025.
- Another ITOG study evaluated Cabozantinib combined with nivolumab and ipilimumab (NCT03914300) in patients previously treated with VEGFR-targeted agents. Its completion is expected in early 2026.
- A randomized Phase II study (NCT02393690) assessed whether Selumetinib could restore iodine uptake in RAI-avid yet refractory metastatic disease. Final data are expected imminently.
- The caboNivoIpi trial (NCT01811212) evaluated Cabozantinib monotherapy as salvage therapy, showing encouraging disease control rates in TKI-resistant settings.
- The LIBRETTO-001 basket trial of Selpercatinib in RET fusion-positive thyroid cancers demonstrated an overall response rate (ORR) of ~79%, with a median progression-free survival (PFS) of 20 months.
- Future studies include neoadjuvant protocols evaluating Selpercatinib before surgery in RET-altered tumors, as well as novel agents targeting PI3K/mTOR pathways and additional multi-kinase inhibitors in phase II trials.

Furthermore, a phase II neoadjuvant trial using patient-derived organoids (PDOs) for drug sensitivity guided individualized therapy in locally advanced thyroid cancer (including DTC). An objective response rate (ORR) of 32.7% was achieved, and 34.5% of patients reached resectable status following treatment based on in vitro testing [138].

Meta-analytic data have highlighted the promise of targeted therapies, demonstrating significant improvements in PFS and overall survival, with risk ratios favoring enhanced disease control across multiple agents, including Lenvatinib, Sorafenib, and Apatinib [139].

Together, these trials illustrate a shift toward precision-guided therapeutic strategies in DTC, aligning genetic alterations (such as RET, BRAF, and NTRK) with targeted agents and adaptive treatment frameworks. They lay the groundwork for future personalized pathways that integrate molecular diagnostics, organoid testing, neoadjuvant therapy, and combination therapy in clinical practice.

### 6.4. Vision for a Fully Personalized Care Pathway

The future of DTC management envisions a seamlessly integrated care pathway where molecular diagnostics, imaging, and patient-centered decision-making converge to deliver truly personalized treatment. A hallmark of this vision is real-time integration of genomic, transcriptomic, and proteomic data with advanced imaging, risk models, and patient-reported outcomes. For example, AI-enhanced ultrasound and cytology tools (validated in studies where AI assistance increased diagnostic accuracy and reduced unnecessary biopsies) can complement molecular profiling to guide early intervention strategies (sensitivity 0.95, AUC ≈ 0.75; unnecessary biopsy rate reduced by ~27%) [131,140].

Future clinical trials(n basket, umbrella, or adaptive designs)will dynamically match patients to therapies based on molecular features, enabling real-time adjustments to treatment arms and endpoints. These protocols permit multi-arm, multi-stage adjustments, amplify statistical power, and support biomarker-driven treatment allocation [141]. Additionally, neoadjuvant biomarker-based trials (e.g., organoid-guided therapy or tyrosine kinase inhibitors targeting specific alterations) lay the groundwork for integrating functional testing into the care continuum, refining surgical decision-making, and tailoring treatment intensity.

Central to this vision are adaptive, patient-centric trials. In these ongoing data, clinical, molecular, imaging, and patient-reported information are used for therapeutic decisions. This approach supports early treatment escalation, sparing low-risk patients and aligning care with individuals’ biology. Supported by robust multi-disciplinary collaboration and real-world evidence platforms, the fully personalized care pathway aims to minimize overtreatment while optimizing long-term outcomes and quality of life in DTC patients.

## 7. Conclusions

DTC management is being reshaped by the integration of molecular diagnostics and personalized therapeutics. Key advances with consensus in clinical practice include the use of BRAF and TERT mutations for prognostic refinement, the adoption of validated molecular panels (Afirma GSC, ThyroSeq v3, MPTX, ThyroidPrint) to guide surgery in indeterminate nodules, and the introduction of selective RET and NTRK inhibitors for advanced RR disease. The 2025 ATA guidelines now incorporate molecular testing into risk stratification and management recommendations, further underscoring its clinical relevance. Despite regional disparities in access and challenges related to cost and infrastructure, these tools are progressively shaping daily practice. Looking forward, technologies such as liquid biopsy, artificial intelligence, and multi-omics integration are expected to consolidate a fully personalized pathway for thyroid cancer care.

## Figures and Tables

**Table 2:** Common Adverse Events of TKIs in RR DTC and Management Strategies.

Drug [Ref]	Key Adverse Events (≥Grade 2)	Monitoring	Management
Sorafenib [86]	HFSR, rash, diarrhea, hypertension, fatigue, elevated liver enzymes	BP monitoring weekly (first 8 weeks); dermatologic evaluation	Emollients, urea-based creams for HFSR; loperamide for diarrhea; antihypertensives for BP control; temporary dose reduction for persistent ≥G2 AEs
Lenvatinib [85,109]	Hypertension, diarrhea, proteinuria, weight loss, fatigue, mucositis	BP monitoring weekly (first 6–8 weeks); urine protein every 4–6 weeks	ACE inhibitors/CCBs for hypertension; dietary modification and loperamide for diarrhea; dose interruption for proteinuria >2 g/24 h
Cabozantinib [110]	Diarrhea, mucositis, hand–foot syndrome, hypertension, thromboembolic events	Regular BP checks; dental exams for mucositis	Mouth rinses (salt/soda) for mucositis; HFSR prophylaxis with moisturizers; anticoagulation assessment if thrombotic risk

BP: Blood pressure, HFSR: Hand–foot skin reaction, ACE: Angiotensin-Converting Enzyme inhibitors.

## Data Availability

Not applicable.
